# Impact of the COVID-19 pandemic on the research activities of UK ophthalmologists

**DOI:** 10.1038/s41433-022-02293-y

**Published:** 2022-10-31

**Authors:** H. D. J. Hogg, L. Low, J. E. Self, Royal College of Ophthalmologists’ Academic and Research Subcommittee, Louise Allen, Denize Atan, Rupert R. A. Bourne, Andrew D. Dick, Paul J. Foster, Richard P. Gale, Christopher J. Hammond, Roly Megaw, Mariya Moosajee, Sobha Sivaprasad, J. S. Rahi

**Affiliations:** 1 Population Health Science Institute, Newcastle University, Newcastle upon Tyne, UK; 2 Newcastle Eye Centre, Newcastle upon Tyne Hospitals NHS Foundation Trust, Newcastle upon Tyne, UK; 3 National Institute for Health Research Biomedical research Centre, Moorfields Eye Hospital, London, UK; 4 Institute of Aging and Inflammation, University of Birmingham, Birmingham, UK; 5 University Hospitals Birmingham NHS Foundation Trust, Birmingham, UK; 6 Southampton General Hospital, Southampton, UK; 7 School of Clinical ad Experimental Sciences, University of Southampton, Southampton, UK; 8 Great Ormond Street Institute of Child Health, University College London, London, UK; 9 Great Ormond Street NHS Foundation Trust, London, UK; 10 Institute of Ophthalmology, University College London, London, UK; 11 Cambridge University Hospitals NHS Foundation Trust, Cambridge, UK; 12 Bristol Eye Hospital, University Hospitals Bristol & Weston NHS Foundation Trust & Translational Health Sciences, University of Bristol Medical School, Bristol, UK; 13 Cambridge University Hospitals NHS Trust, Cambridge & National Specialty Lead Ophthalmology, NIHR Clinical Research Network, Cambridge, UK; 14 UCL-Institute of Ophthalmology, London, UK; 15 University of Bristol, Bristol, UK; 16 Hull and York Medical School, University of York, York, UK; 17 York and Scarborough Teaching Hospitals NHS Foundation Trust, York, UK; 18 Section of Ophthalmology, King’s College London, London, UK; 19 Guys and St Thomas’ NHS Foundation Trust, London, UK; 20 MRC Human Genetics Unit, University of Edinburgh, Edinburgh and Princess Alexandra Eye Pavilion, NHS Lothian, Edinburgh, UK

**Keywords:** Scientific community, Medical research

## Abstract

**Background:**

The COVID-19 pandemic has impacted negatively on many areas of biomedical research and there is concern that academic recovery will take several years. This survey aimed to define the impact of the COVID-19 pandemic on UK ophthalmologists’ research activities and understand the implications for recovery.

**Methods:**

An online survey comprising multiple choice and free-text questions was designed, piloted and then distributed to Royal College of Ophthalmologists (RCOphth) members in January 2021. Respondent characteristics, research expectations and experiences through the pandemic were captured. Descriptive and comparative statistics were applied to quantitative data alongside content analysis of qualitative data.

**Results:**

In total, 148 respondents (3.7% of RCOphth membership) comprised 46 trainees (31.1%), 97 consultants (65.5%) and 5 SAS doctors (3.4%); 54 had clinical-academic roles (36.5%) and 65/94 (69.1%) ophthalmologists with fully clinical posts identified as research-active. Of 114 research-active respondents, 104 (91.2%) reported an impact on their research from COVID-19; negative impacts included loss of research time (*n* = 69), research delays (*n* = 96) and funding shortfalls (*n* = 63). Content analysis identified five common themes; type of research activity, clinical demands, institutional challenges, COVID-19 alignment and work-life balance.

**Conclusions:**

UK ophthalmology research has been adversely impacted by the pandemic. A substantial proportion of UK ophthalmologists are research active, but 20.4% of those surveyed felt that the pandemic had made research less attractive. Strategic steps must be taken to nurture UK ophthalmologists’ engagement with research, especially for those who currently do no research, if the profession is to align itself with the Government vision of ‘Research for All’.

## Introduction

As the pandemic caused by SARS-CoV-2 infection developed within the UK, significant adverse impacts on biomedical research and clinical academia materialised rapidly from March 2020 due to a combination of events [1]. Firstly, research facilities and academic institutions shut down laboratories and clinical trials to all but COVID-19 related research. Secondly, patient-facing research requiring either recruitment of participants through National Health Service (NHS) care or access to its healthcare facilities such as imaging was interrupted. Finally, research time was lost to clinical duties as many clinical academics were re-deployed to support frontline NHS services. This affected all career stages, but particularly academic trainees whose research training was consequently interrupted. Centralised attempts to mitigate all these impacts on clinical research were made through the National Institute for Health Research (NIHR) Restart Framework, first published in May 2020 with five subsequent revisions up to January 2021 [2].

Evidence of the immediate and longer term adverse impacts on biomedical research and clinical academia grew alongside rapid learning about the potential positive impacts of some of the adaptions to working in the pandemic [3–5]. Wide-ranging impacts on institutions, researchers and clinicians have been reported from several geographical and specialty contexts, but not for vision and eyes research or more specifically for ophthalmologists undertaking research in the UK [6–18]. To address this evidence gap and enable the development of ophthalmology-specific mitigation strategies (if required) the Academic and Research Subcommittee of the Royal College of Ophthalmologists (RCOphth) sent a survey to all UK ophthalmologists via the RCOphth membership list to ascertain their views and experiences of the impact of the pandemic on their research activities. The survey aimed to quantify and characterise the impact of the COVID-19 pandemic on research activities and future plans and to understand the implications for the recovery of ophthalmic research.

## Methods

### Survey design

An anonymous online survey was designed and piloted by members of the RCOphth Academic and Research Subcommittee to capture information from all ophthalmologists at all career stages, whether they held clinical and academic research posts or solely clinical posts. The survey comprised questions to characterise the respondents’ roles and elicit any positive and negative impacts of the pandemic on their pre-existing research activities and their plans for future research activities. The survey was designed using Microsoft Forms (Microsoft Corp. Redmond, WA. 2020) and used a branching design to target and therefore minimise the number of questions that each respondent had to answer, with no respondent having to answer more than 18 questions (Supplementary material 1).

### Survey distribution

The survey was not distributed individually, in keeping with RCOphth policies. Instead, the link to the survey was publicised through RCOphth communication channels to the whole membership and via the RCOphth Ophthalmic Trainees Group and specific subspecialty groups, e.g. paediatric ophthalmologists. As the survey was not distributed individually and as responses were anonymised, no individual reminders were sent.

### Analysis

Quantitative data were analysed in SPSS v.24 (IBM Corporation, New York, USA) to facilitate data visualisation, descriptive statistics and Chi-squared tests for categorical comparative testing. Responses to the five free text questions in the survey were coded by the type and grade of their contributor and entered into NVivo Release 1.2 (426) (QSR International, MA, USA). Here, content analysis was used to inductively and iteratively generate themes supported by the full authorship team.

## Results

### Quantitative analysis

One-hundred and forty-eight respondents (Table 1) of the total 4029 RCOphth membership who were notified of the survey (3.7% response rate) completed the survey at a median of 1.2 months (inter-quartile range 0.7, 2.8) after the start of the 3rd UK lockdown, on January 6th 2021, taking a median time of 2 min and 34 s (IQR 1 min 23 s, 3 min 56 s). The mean survey completion time was 4 min 25 s.

**Table 1:** Table showing composition of ophthalmologist respondents by clinical grade and academic post.

		Count (*n* = 148)	% of cohort
Trainee	Non-academic post	26	17.6%
	Academic post	20	13.5%
Consultant	Non-academic post	65	43.9%
	Academic post	32	21.6%
SAS	Non-academic post	3	2.0%
	Academic post	2	1.4%

*SAS* Specialty doctors and Associate Specialists.

Of the 54/148 respondents (36.4%) who reported holding academic posts, 29/54 (53.7%) and 14/54 (25.9%) had the NHS or a higher education institution (HEI) as their substantive employer respectively, with 11/54 (20.4%) in a temporary ‘out of programme’ (OOP) arrangement. Ophthalmology specialty trainees cumulatively represented 12/19 different UK deaneries. 10 respondents (23.8%) being in the London deanery, which hosts 151 of the 699 ophthalmology specialty trainees (21.6%) registered as of February 2022. The 46 trainee respondents represented a 6.6% response rate among specialty trainees.

**Fig. 1:**
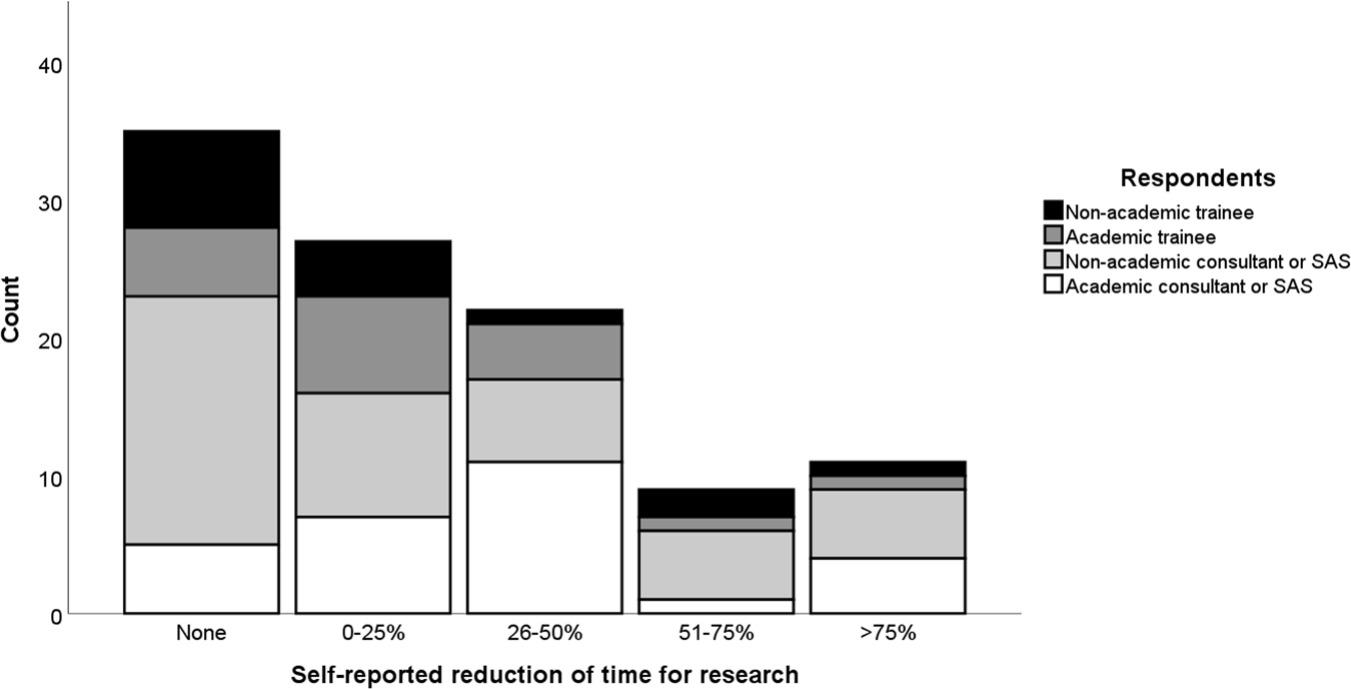
Research time reduction by grade and type of respondentsʼ post. A histogram showing frequency of self-reported estimates of proportional loss of personal research time due to the SARS-CoV-2 pandemic. SAS specialty doctors and associate specialists.

**Fig. 2:**
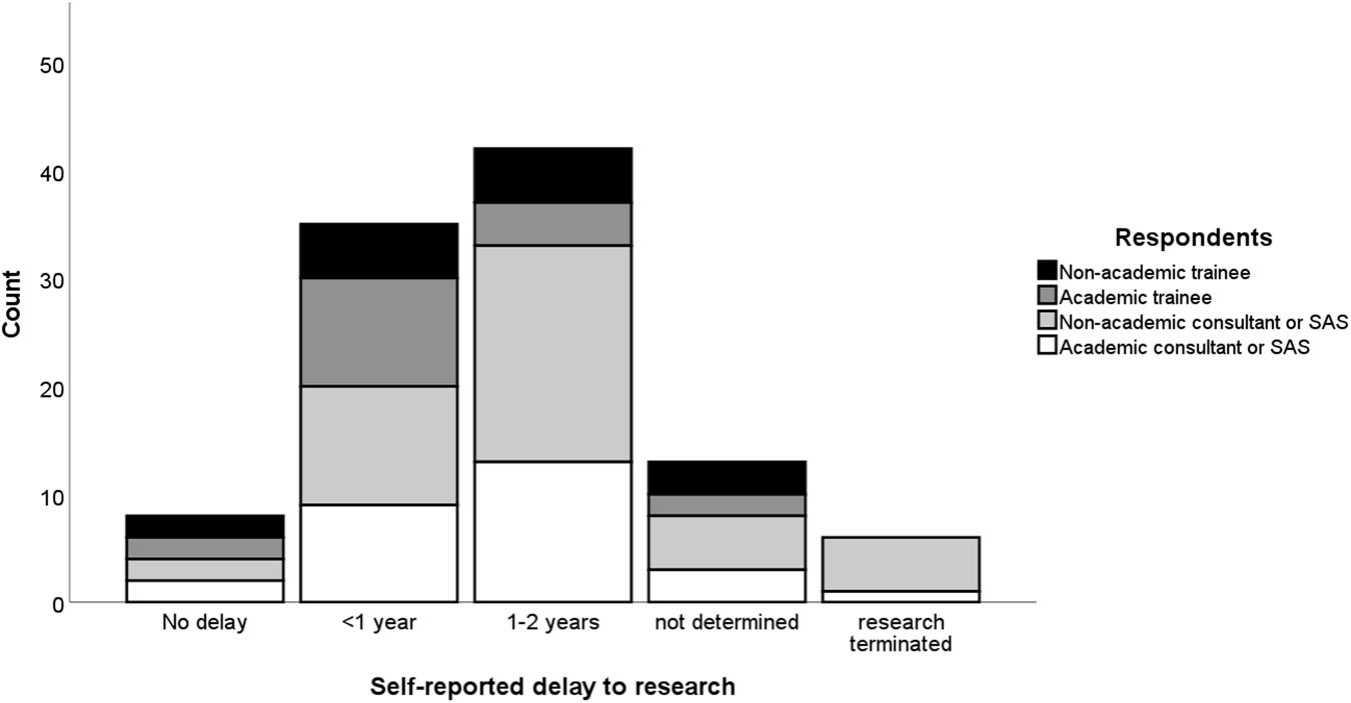
Delay to research by grade and type of respondentsʼ post. A histogram showing frequency of self-reported estimates of delay to research projects due to the SARS-CoV-2 pandemic. SAS specialty doctors and associate specialists.

**Fig. 3:**
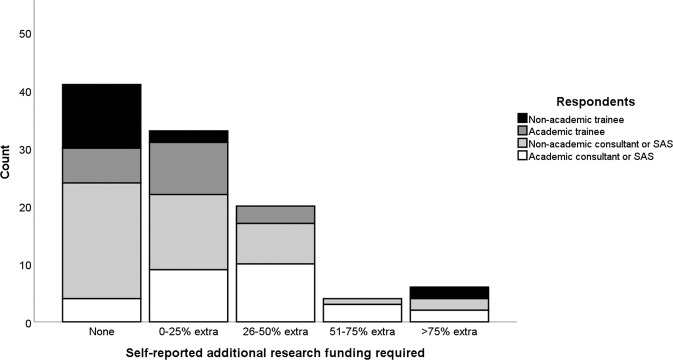
Additional research funding required by grade and type of respondentsʼ post. A histogram showing frequency of self-reported estimated of additional research funding requirements due to the SARS-CoV-2 pandemic. SAS specialty doctors and associate specialists.

Most respondents who did not have academic posts reported engaging in some form of research activity, including 19/26 ophthalmology trainees (73.1%) and 46/68 Specialty doctors and Associate Specialists (SAS) or consultant ophthalmologists (67.6%). Fewer than a quarter (34/148, 22.9%) of all respondents said they did no research. The large majority (91/148, 79.8%) of those who did do research reported an overall negative impact of the pandemic on their research activities. A negative impact was more commonly reported by research-active SAS and consultant grades than trainee grades, 64/75 (85.3%) versus 27/39 (69.2%) (*p* = 0.04). A negative impact was more commonly reported by those holding academic posts than those who did not, 43/49 (87.8%) vs 48/65 (73.8%) (*p* = 0.07). 10/114 research active respondents (8.8%) reported no impact to their research from the COVID-19 pandemic. Of 104 respondents reporting some impact from the pandemic on their research. Overall, 91 (87.5%) experienced a negative impact and 13 (12.5%) experienced the impact positively. 69/104 (66.3%) felt that the time they had available for research had been decreased (Fig. 1). Of these same respondents, 96/104 (92.3%) reported some degree of delay or termination of their research projects (Fig. 2) and 63/104 (60.6%) reported a need for further funding to maintain their planned research activities (Fig. 3). The reasons given for the research impact experienced were varied, but most commonly related to capacity of NHS institutions (Table 2). When asked whether the pandemic had made research more or less attractive to them 29/46 (63.0%), 11/46 (23.9%) and 6/46 (13.0%) trainees said it had no impact, was more attractive or less attractive respectively, compared to 59/101 (58.4%), 18/101 (17.8%) and 24/101 (23.8%) SAS and consultant ophthalmologists (*p* ≥ 0.05 for differences).

**Table 2:** Responses from 104 respondents reporting impact on their research from the COVID-19 pandemic when asked why they experienced the impact on their research.

	Limits to research experienced by respondents; n (%)				
Respondent group	Clinical work	NHS research capacity	HEI capacity	Loss of funding	Other
Trainees (*n* = 46)	11 (23.9)	13 (28.3)	20 (43.5)	4 (8.7)	6 (13.0)
Consultants and SAS (*n* = 102)	35 (34.3)	51 (50.0)	23 (22.5)	17 (16.7)	18 (17.6)
Fully clinical posts (*n* = 94)	20 (21.3)	36 (38.3)	17 (18.1)	7 (7.4)	13 (13.8)
Clinical academic posts (*n* = 54)	26 (48.1)	28 (51.9)	26 (48.1)	14 (25.9)	11 (20.4)

*NHS* National Health Service, *HEI* Higher Education Institution.

### Content analysis

The responses to the free text questions in the survey and their analysis (Supplementary material 1), identified five distinct themes.

#### Impacts related to type of research activity—46 supporting comments

The nature of activities required for a given research project, as well as how the focus of the research could be related to the COVID-19 pandemic clearly impacted its viability during the pandemic. In particular, clinical research and laboratory science appeared to suffer due to reduced patient access, additional risks from attending healthcare premises and the temporary closure of many HEI laboratories. Meanwhile, observational research seemed to prosper along with research activities that could continue or were made more efficient by the culture shift toward using virtual platforms.

*‘I don’t undertake laboratory research. Fewer competing activities outside of work time and less hectic elective activity allowed more time to undertake research.’ —non-academic trainee*

*‘Easier to arrange supervision meetings and PPI [patient and public involvement] virtually’- academic trainee*

#### Clinical demands—52 supporting comments

During the pandemic, the demands experienced by respondents from clinical service varied. On one hand, the cancellation of elective services left some ophthalmologists with more time to pursue research whilst others found themselves or members of their research team redeployed to other services.

‘*I have had more time for research as private work stopped and NHS operating largely stopped’– non-academic consultant*

‘*Redeployment of research staff has had [a] negative impact on morale, uncertainty about ‘restart’ has increased worries about [the] future.’- academic consultant*

There was a sense that the workload associated with the clinical backlog would compromise research activities into the future once elective work carefully resumes. These sentiments arose mainly from non-academic consultant and SAS respondents who often felt the invasion of their protected administration time in which they undertook research was inevitable.

#### Consequence from institutional challenges—26 supporting comments

Many respondents’ research efforts were frustrated by institutional barriers that were not present outside of the pandemic. Funding opportunities from public, private and charity sectors were all reported to be negatively impacted. In some instances, funding had been secured but respondents were unable to implement the funded research as the necessary human and physical resources were not available. For the most part, funders appeared unable to mitigate these strains, though there were examples of extension to awards which lessened the impact on individuals and their work.

*‘Reduced funding available from smaller funding bodies and research charities- this has been catastrophic with many charities not being able to generate adequate funds to provide the usual level of funding and therefore declining grant applications which would have been successful in other years.’ – academic trainee*

#### Aligning research to COVID-19–20 supporting comments

Two separate consequences arose from the wider research community’s shift in focus to research to support the response to the pandemic. Respondents found that pre-existing research projects outside of this scope were de-prioritised by funders and host institutions. However, other respondents who devised new research projects that directly or indirectly addressed the threats posed by the pandemic reported successes in research delivery and dissemination.

‘*I’ve been able to fast track a study to validate VA [visual acuity] self-testing at home and gain approval in the hospital and community for a pilot.’ – non-academic consultant*

*‘Supporting engineering staff were diverted to covid related projects’ – academic trainee*

#### Work-life balance—13 supporting comments

Five respondents suggested that the lack of competing demands for their spare time, led them to complete more research activities outside of their contractual working hours. This was not universal as some clinical academics who were doing academic work from home, found that a busy home life involving home schooling or childcare demands limited their productivity. Aside from the impact of clinical work, there was also evidence that the pandemic’s impact on HEIs led some senior clinical academics to suffer a great deal of pressure from their academic responsibilities.

‘*I am averaging 100–120* *hs a week working time, but the university is still putting more and more chores back on us academics.’- academic consultant*

## Discussion

These survey data illustrate the substantial negative impact of the SARS-CoV-2 pandemic on research within UK ophthalmology. However, it is encouraging to see only a minority of respondents felt that, as a result research has become less attractive to them, and indeed that some respondents felt more enthusiastic about undertaking research in future. Alongside this, many enforced changes to ways of working, such as virtual meetings and greater flexibility during pauses in face-to-face clinical tasks appeared to have improved the ease and efficiency of certain research activities. These observations mirror the substantial rises in the rate at which manuscripts have been submitted to academic journals during the pandemic [19–21]. Aside from taking advantage of some aspects of more flexible working, a small number of ophthalmologists were also able to reframe their activities to include an aspect addressing questions specifically related to the COVID-19 pandemic. This enabled these individuals to continue or expand their research, but it was not the norm. Finally, it is notable that the majority of respondents to this survey were research active and were not in formal clinical academic posts.

To our knowledge this is the first assessment of the impact of the pandemic on UK ophthalmic research based on responses from ophthalmologists undertaking the research themselves. As such it provides otherwise unavailable information necessary for planning activities to mitigate the impact of the pandemic, including advocacy work, and identifying where specific approaches may be required in ophthalmology. Nevertheless, there are limitations to this study. Firstly, the measurement of impact is based on individual respondent reports rather than objective data. This issue overlies potential responder bias, whereby the small portion of the RCOphth membership that responded to the survey were likely to be more concerned about the COVID-19 pandemic than non-responders. Surveys conducted with RCOphth membership generally achieve below 24% response rate. Mitigating the present study’s low response rate through repeated invitations to non-responding RCOphth members was not permitted. This means it is not possible to draw definitive quantitative conclusions, for example the scale of the time delay to research, but the survey does afford an understanding of the real-world challenges that ophthalmologists face. These insights are valuable, particularly as more objective measures of research impact are often unavailable, such as the number of clinical redeployments experienced by academic trainees which the study team sought from the NIHR, but were not available. It is also true that whilst certain sub-populations are underrepresented or overrepresented, e.g., non-research active members or academic trainees respectively, there were sufficient responses to facilitate a meaningful insight into each main group’s perspective. A further limitation of the study design is the lack of denominators for all respondent characteristics, which were not available from the RCOphth due to data protection issues. This limits the granularity with which response rate and selection bias can be assessed. Similarly, the relatively novel requirements for this study meant that although the survey was developed and trialled iteratively among the research team, it lacked a rigorous testing and validation process which may influence the data quality. The average completion time was short and suggests respondent burden was not high.

The findings of our survey show that the complex adverse impact on UK ophthalmology research includes delayed delivery of funded research and loss of personal research time. Projects being put on hold whilst their funding windows continue to run to schedule also limited the resources investigators had available to some investigators. Ophthalmology is a specialty that relies disproportionately heavily on funding from small specialist charities. It will therefore be particularly hard hit by the significant drop in income for UK medical charities that is attributable to the pandemic, with the predicted 40% decrease in their medical research spending power, creating a shortfall of £310 million and an anticipated 4.5-year period before their medical research spend recovers to normal levels [22]. This will cast a long shadow over vision and eyes research in the UK. Advocacy by the RCOphth will be more important than ever.

In the midst of the second acute wave of the pandemic, the RCOphth Academic Subcommittee published in ‘Mitigating the impact of COVID-19 on Academic Ophthalmology and Research’ its guidance and recommendations for actions by ophthalmologists, research funders and NHS bodies and other stakeholders [23]. The findings of this study allow those recommendations to be developed further. For example, to address the evidence provided by respondents of the challenge to work life balance in juggling clinical commitments, research activities and broader academic responsibilities and duties. This need is highlighted by evidence from other specialties of burnout and a drop in productivity, both from absenteeism and “presenteeism” [24].

The RCOphth Academic and Research Subcommittee’s recommendations, whilst focusing on the impact of the pandemic, naturally also took account of the pre-existent challenges faced by vision and eyes research and concerns about the decline in senior clinical academic workforce [23]. The results of this survey have added to what is already anticipated about impacts of the pandemic on vision and eyes research by providing broader insights into research participation by UK ophthalmologists and the barriers they perceive to be present. This is particularly timely, given the publication in 2021 of the UK Government’s ‘Future of Clinical Research Delivery’ policy paper and its subsequent implementation plan for the year ahead [25]. The central theme is that clinical research is the core business of all clinicians in the NHS and this builds on the adoption of metrics relating to research within the Care Quality Commission’s Well-led framework and the commitment to this direction of travel from regulators and research funders [26]. A number of medical specialities are already significantly ahead in embracing and promoting the culture change required by “Research by All” [27, 28]. Facilitators include new funding streams to support SAS and consultants to become principal investigators, regardless of their research background [29, 30]. Learning from the challenges of conducting research during the height of the pandemic is relevant to ensuring that appropriate priority is given to protecting the time and space clinicians need to be able to engage properly in research. This engagement may take many forms, be it supporting trainees to undertake projects or being a local principal investigator on a large multicentre trial.

A new ophthalmology specialist training curriculum comes into effect soon which sees a major change to enhance skills, acquired experience and attitudes to research [31]. The delivery of this new curriculum will influence trainees’ careers as consultants, but also the future shape of ophthalmic clinical research in the NHS. It will also determine whether the UK remains world-leading in vision and eyes research and in training vision and eyes scientists. The responses from trainees participating in our survey are therefore timely and aptly identify the vital role that everyone involved in delivering training, including the RCOphth, will play in making the new curriculum a success. Together, these contributors will create a research-engaged and research-enabled clinical workforce, so that as a specialty ophthalmology can align with the Government’s ambitions for research in the NHS.

The future of clinical research delivery in the NHS also depends on the perspectives of patients and potential participants. Whilst our survey does not offer insights into this, it is heartening to note from other studies that participants have remained willing to engage during the pandemic [32]. To some extent this willingness is contingent on their trust in the Government and healthcare professionals, as well as COVID-19 risk mitigation measures such as testing for participants and additional efforts to shorten visit length and contact with other individuals [33]. These observations were echoed in a specific study of participants with visual disabilities [34].

The RCOphth Academic and Research Subcommittee thanks all ophthalmologists who participated in the survey reported here. We will draw on the findings as baseline intelligence to support the development of strategy and policy which protects and nurtures the research culture of UK ophthalmologists.

## Summary

### What was known before

- The COVID-19 pandemic has been reported to impact biomedical research in many contexts, often negatively if not directly related to the pandemic itself. In the UK, this has come at a time where government strategy is to promote and support clinicians’ research activities and foster a ‘research for all’ culture within the NHS.

### What this study adds

- Many UK ophthalmologists are research active, despite being in full time clinical posts. Most respondents experienced a negative impact on their research activities in terms of project delay or abandonment and access to funding.

## Supplementary information

Supplementary material 1

Supplementary Material

## Data Availability

As part of the RCOphth’s work with it’s membership, public availability of raw data was outside of scope and ethical approval to do so was not sought.

## References

[1] National Institute for Health Research. DHSC issues guidance on the impact of COVID-19 on research funded or supported by NIHR 2020 [16/03/2020] https://www.nihr.ac.uk/news/dhscissues-guidance-on-the-impact-on-covid-19-on-research-funded-or-supported-by-nihr/24469

[2] National Institute for Health Research. A framework for restarting NIHR research activities which have been paused due to COVID-19 2020.

[3] van Dorn A (2020). COVID-19 and readjusting clinical trials. Lancet.

[4] Byrne JA, Carpenter JE, Carter C, Phillips K, Braye S, Watson PH (2021). Building research support capacity across human health biobanks during the COVID-19 pandemic. Biomarker Insights..

[5] Lorusso D, Ray-Coquard I, Oaknin A, Banerjee S (2020). Clinical research disruption in the post-COVID-19 era: Will the pandemic lead to change?. ESMO Open..

[6] Subramain M, Wangui-Veryy JM, Sprenger KJ, Comellas AP, Barlow PB (2021). Impact of COVID-19 on clinical research units (CRUs). J Clin Transl Sci..

[7] Sarkar S, Aggarwal R (2020). Covid-19 Pandemic: A spoiler for health research. Natl Med J India.

[8] Burchill E, Lymberoupoulos E, Menozzi E, Budhdeo S, Mcllroy JR, Macnaughtan J (2021). The unique impact of COVID-19 on human gut microbiome research. Front Med..

[9] De B, Kaiser KW, Ludmir EB, Yeboa DN, Tang C, Hoffman KE (2021). Radiotherapy clinical trial enrollment during the COVID-19 pandemic. Acta Oncologica..

[10] Ehrlich H, McKenney M, Elkbuli A (2021). The impact of COVID-19 pandemic on conducting emergency medicine clinical research. Am J Emerg Med.

[11] Joshi D, Hill N, Hruby A, Viswanathan S, Ingo C, Roth H (2021). Stakeholder perspectives on engaging with cerebral palsy research studies after onset of COVID-19 in the United States. Arch Phys Med Rehabilitation..

[12] Ma X, Luc JGY, Vervoort D (2020). Moving forward: Ensuring quality research in vascular surgery during COVID-19. J Vasc Surg.

[13] McLaughlin RA, Madigan V, O’Grady M, Korpanty G, Coate L (2020). The impact of the COVID-19 pandemic on oncology clinical trials. Ir Med J..

[14] Ndumele A, Park KU (2021). The impact of COVID-19 on national clinical trials network breast cancer trials. Curr Breast Cancer Rep..

[15] Sundaragiri K, Panda A (2020). Effect of COVID19 on oral research in Indian scenario: An observation. J Oral Maxillofac Pathol.

[16] Tran NN, Tran M, Lopez J, Woon J, Nguyen J, Brecht ML (2021). Impact of COVID-19 on Pediatric Clinical Research. J Pediatr Nurs.

[17] Castro-Sánchez E, Russell AM, Dolman L, Wells M (2021). What place does nurse-led research have in the COVID-19 pandemic?. Int Nurs Rev.

[18] Maxton F, Darbyshire P, Thompson DR (2021). Research nurses rising to the challenges of COVID-19. J Clin Nurs.

[19] Favorito LA (2020). Increase in submissions to International Brazilian Journal of Urology during Covid-19 quarentine. Int Braz J Urol.

[20] Chillakuru YR, Gerhard EF, Shim T, Selesnick SH, Lustig LR, Krouse JH (2022). Impact of COVID-19 on otolaryngology literature. Laryngoscope..

[21] Lee JE, Mohanty A, Albuquerque FC, Couldwell WT, Levy EI, Benzel EC (2020). Trends in academic productivity in the COVID-19 era: analysis of neurosurgical, stroke neurology, and neurointerventional literature. J Neurointerv Surg..

[22] Association of Medical Research Charities. COVID-19: The risk to AMRC charities. 2020; Available from: https://www.amrc.org.uk/covid-19-the-risk-to-amrc-charities.

[23] The Royal College of Ophthalmologists. Mitigating the impact of COVID-19 on Academic Ophthalmology and Research. 2020; Available from: https://www.rcophth.ac.uk/2020/07/mitigating-the-impact-of-covid-19-on-academic-ophthalmology-and-ophthalmic-research/.

[24] Sharma MK, Anand N, Singh P, Vishwakarma A, Mondal I, Thakur PC (2020). Researcher burnout: An overlooked aspect in mental health research in times of COVID-19. Asian J Psychiatry.

[25] The Department of Health and Social Care. The Future of UK Clinical Research Delivery: 2021 to 2022 Implementation Plan. 2021.

[26] Care Quality Commission. Inspection framework: NHS trusts and foundation trusts. 2020.

[27] Royal College of Physicians. Delivering research for all: expectations and aspitations for the NHS in England. 2019.

[28] The Royal College of Ophthalmologists. The Royal College of Ophthalmologists endorses call for ‘Research for All’. 2019; Available from: https://www.rcophth.ac.uk/2019/04/the-royal-college-of-ophthalmologists-endorses-research-for-all/.

[29] National Institute for Health Research. Associate Principal Investigator Scheme. 2021; Available from: https://www.nihr.ac.uk/health-and-care-professionals/career-development/associate-principal-investigator-scheme.htm. 10.1136/archdischild-2024-327675 39904588

[30] National Institute for Health Research. Greenshoots Scheme. 2021; Available from: https://local.nihr.ac.uk/documents/greenshoots-scheme/28594.

[31] The Royal College of Ophthalmologists. Consultation on proposed OST Curriculum. 2021; Available from: https://www.rcophth.ac.uk/2021/04/consultation-curriculum/.

[32] Gobat N, Butler CC, Mollison J, Francis NA, Gal M, Harris V (2019). What the public think about participation in medical research during an influenza pandemic: an international cross-sectional survey. Public Health..

[33] Padala PR, Jendro AM, Padala KP (2020). Conducting clinical research during the COVID-19 pandemic: Investigator and participant perspectives. JMIR Public Health Surveillance.

[34] Kim HN, Sutharson S (2021). Concerns and needs of research participants with visual disabilities amid the COVID-19 pandemic. Theoret Issues Ergonomics Sci..

