# Supplementary material for: Impact of the COVID-19 pandemic on the research activities of UK ophthalmologists
Source: Eye (Lond). 2022 Oct 31;37(10):2089–94. doi: 10.1038/s41433-022-02293-y (PMC9628368; doi:10.1038/s41433-022-02293-y)
Supplement: Supplementary file 1 — Supplementary material 1 [file 41433_2022_2293_MOESM1_ESM.pdf]

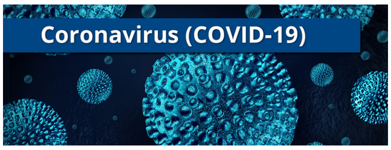

COVID-19 - Impact on research

An anonymous survey to scope the impact of COVID-19 on research activities for research-active UK Ophthalmologists

* Required

Why are we doing this survey?

The COVID-19 pandemic has impacted on all parts of our lives and work as UK based Ophthalmologists. In a time of such pressure on Ophthalmologists' time, there has been concern about the impact on ophthalmic research. From funding and staffing to regulatory hurdles and time-pressures, there is currently no data on how the pandemic has impacted Ophthalmic research. In order for the RCOphth to help support and promote UK based Ophthalmology research, we would like to gather some basic data through this survey. Thank you for your help.

About you

1. Which of the following would best describe your current job? *

- ◯ Ophthalmic trainee of any type
- ◯ Substantive NHS consultant
- ◯ Substantive NHS consultant + honorary academic contract
- ◯ Substantive NHS specialty doctor
- ◯ Substantive NHS specialty doctor + honorary academic contract
- ◯ Substantive Clinical academic with honorary NHS consultant contract
- ◯ Post CCT but no consultant post

2. Which of the following best describes your training post? *

- ◯ ACF currently doing an OOPR
- ◯ ACF currently NOT doing an OOPR
- ◯ ACL currently doing an OOPR
- ◯ ACL NOT currently doing an OOPR
- ◯ Non-academic clinical trainee

3. Which of the following best describes your training stage? *

- ◯ ST1
- ◯ ST2
- ◯ ST3
- ◯ ST4
- ◯ ST5
- ◯ ST6
- ◯ ST7
- ◯ LAT
- ◯ FY3

4. Are you a member of your regional ophthalmology trainee research network? *

- ◯ Yes
- ◯ No

5. Please specify which region *

6. Are there any other reasons that you are not a member of your regional ophthalmology trainee research network?

About your research

7. Has the COVID-19 pandemic affected your research activities (either positively or negatively)? *

   - ◯ Yes
   - ◯ No

8. The COVID-19 pandemic has had no impact on my research activities because: *

   - ◯ I don't do any research
   - ◯ Impact has been avoided

9. If COVID-19 impact on your research has been avoided? Please describe how (as briefly as possible): *

10. Overall, has the COVID-19 pandemic had a net positive or negative impact on your research activities overall? *

    - ◯ Positive
    - ◯ Negative

11. Please use keywords to describe any positive impacts from COVID-19 on your research

12. For any negative impacts from COVID-19 on your research, please select all that apply from the below: *

- [ ] Loss of funding
- [ ] Reduced time for research due to increased clinical work for you or your team
- [ ] Reduced capacity or closure of NHS research facilities or support
- [ ] Reduced capacity or closure of higher education/research institute facilities/resources
- [ ] Other
- [ ] None

13. Please describe as concisely as possible any other negative impacts from COVID-19 on your research that were not listed in the previous question.

14. By how much has your research been delayed? *

- ◯ Not at all
- ◯ Research terminated as non longer viable
- ◯ Less than 1 year
- ◯ 1-2 years
- ◯ 2-3 years
- ◯ 3-4 years
- ◯ Longer than 4 years
- ◯ Not yet determined

15. As an overall percentage, how much additional funding (estimate) would be required to complete your planned research due to the pandemic? *

- ◯ None
- ◯ 0-25% of funding
- ◯ 26-50% of funding
- ◯ 51-75% of funding
- ◯ More than 75% of funding

16. With regards to any loss of funding you have experienced due to the pandemic, is this permanent or temporary? *

- ◯ Permanent
- ◯ Temporary
- ◯ A mixture of both
- ◯ No loss of funding

17. If the pandemic has reduced the amount of time you have for research, can you estimate by how much overall between March 2020 and the present time? *

- ◯ Not at all
- ◯ 0- 25%
- ◯ 26-50%
- ◯ 51-75%
- ◯ More than 75%

18. What reduction in your time for research do you anticipate from the present time until the end of 2021? *

- ◯ None
- ◯ 0- 25%
- ◯ 26-50%
- ◯ 51-75%
- ◯ More than 75%

19. Why do you think that your time for research will be reduced from the present time until the end of 2021? *

- ☐ Reduced capacity or closure of NHS research facilities or support
- ☐ Reduced capacity or closure of higher education/research institute facilities/resources
- ☐ Expected to use SPA and research time to help address patient waiting lists
- ☐ Loss of funding
- ☐ Other

20. Please describe, as concisely as possible, any other reasons why your time for research may be reduced from the present time until the end of 2022?

21. Has the pandemic made research more or less attractive to you?

- ◯ More
- ◯ Less
- ◯ No difference

Thank you

Thank you for completing the survey. The data will help us to support and promote Ophthalmologists in the UK with regards to research activity.

This content is neither created nor endorsed by Microsoft. The data you submit will be sent to the form owner.

Microsoft Forms
