# Supplementary material for: Impact of the COVID-19 pandemic on the research activities of UK ophthalmologists
Source: Eye (Lond). 2022 Oct 31;37(10):2089–94. doi: 10.1038/s41433-022-02293-y (PMC9628368; doi:10.1038/s41433-022-02293-y)
Supplement: Supplementary file 2 — Supplementary Material [file 41433_2022_2293_MOESM2_ESM.docx]

# Supplementary Material

Supplementary material 1. COVID-19 – Impact on research; An anonymous survey to scope the impact of COVID-19 on research activities for research active UK Ophthalmologists
